# Acute Stress Induces Hyperacusis in Women with High Levels of Emotional Exhaustion

**DOI:** 10.1371/journal.pone.0052945

**Published:** 2013-01-02

**Authors:** Dan Hasson, Töres Theorell, Jonas Bergquist, Barbara Canlon

**Affiliations:** 1 Department of Physiology and Pharmacology, Karolinska Institutet, Stockholm, Sweden; 2 Stress Research Institute, Stockholm University, Stockholm, Sweden; 3 Department of Chemistry – BMC, Analytical Chemistry, Uppsala University, Uppsala, Sweden; University of Regensburg, Germany

## Abstract

**Background:**

Hearing problems is one of the top ten public health disorders in the general population and there is a well-established relationship between stress and hearing problems. The aim of the present study was to explore if an acute stress will increase auditory sensitivity (hyperacusis) in individuals with high levels of emotional exhaustion (EE).

**Methods:**

Hyperacusis was assessed using uncomfortable loudness levels (ULL) in 348 individuals (140 men; 208 women; age 23–71 years). Multivariate analyses (ordered logistic regression), were used to calculate odds ratios, including interacting or confounding effects of age, gender, ear wax and hearing loss (PTA). Two-way ANCOVAs were used to assess possible differences in mean ULLs between EE groups pre- and post-acute stress task (a combination of cold pressor, emotional Stroop and Social stress/video recording).

**Results:**

There were no baseline differences in mean ULLs between the three EE groups (one-way ANOVA). However, after the acute stress exposure there were significant differences in ULL means between the EE-groups in women. Post-hoc analyses showed that the differences in mean ULLs were between those with high vs. low EE (range 5.5–6.5 dB). Similar results were found for frequencies 0.5 and 1 kHz. The results demonstrate that women with high EE-levels display hyperacusis after an acute stress task. The odds of having hyperacusis were 2.5 (2 kHz, right ear; left ns) and 2.2 (4 kHz, right ear; left ns) times higher among those with high EE compared to those with low levels. All these results are adjusted for age, hearing loss and ear wax.

**Conclusion:**

Women with high levels of emotional exhaustion become more sensitive to sound after an acute stress task. This novel finding highlights the importance of including emotional exhaustion in the diagnosis and treatment of hearing problems.

## Introduction

Hearing problems constitute one of the top ten public health disorders in the general population affecting more than 30% of the population when evaluated with self-reported questionnaires [1], [2]. Hearing problems, including hearing loss, difficulties in understanding speech in noise, tinnitus and hyperacusis are primarily caused by damage to the auditory periphery and/or the central auditory system. It is well established that hearing loss and tinnitus are age-related and that men are more often affected than women [3], [4]. It has also been shown that hearing problems are co-morbid with tooth loss, diabetes, psychiatric conditions and cardiovascular disease [5], [6], [7], [8] and associated with socioeconomic status [2]. Despite the enormous numbers of individuals suffering from hearing disorders, the influence of psychosocial factors is largely unknown.

While there is increasing evidence for a relationship between stress and hearing problems its causality is not well-established. In experimental animals, acute stress has been shown to protect the auditory system from a subsequent noise trauma [9]. However, direct evidence for the effects of stress, whether acute or chronic, on human hearing has not yet been directly tested. Recently, it was shown that emotional exhaustion is the variable that is the most strongly associated with tinnitus prevalence, more strongly associated than traditional risk factors such as hearing loss, noise-exposure, smoking and hypertension [10]. It is not known whether stress induces the hearing problems or if stress is a consequence of them. It is plausible that the association is bi-directional, i.e. that hearing problems are stressful and that stress causes increased vulnerability to hearing problems.

Presently, there is a void of studies on the causality of stress exposure and hearing problems in humans. It is not known if individuals with different degrees of emotional exhaustion (EE) will have different auditory sensitivity or if that sensitivity is altered by an acute stress exposure. The hypothesis being tested in this study is that an acute stress exposure will increase auditory sensitivity in individuals with high levels of EE. This would be in line with previous studies showing maladaptive reactions in the direction of increased hypersensitivity [11], [12], [13], [14], [15], [16]. In contrast, the adaptive reaction would result in an increased tolerance or resilience after an acute stress [11], [14], [15], [16]. Over-sensitivity is a common feature in many stress-related disorders [12], [13]. In fact, patients with various stress-related disorders often have a high level of co-morbidity [12], [17], [18], [19], [20] and therefore the concept multi-illness syndrome is often used to define these stress-related conditions. A common symptom in individuals with hearing problems is hyperacusis, which is over-sensitivity and discomfort to normal environmental sounds that are easily tolerated by individuals without hyperacusis [21]. At present, there is no mechanistic explanation for this type of vulnerability.

Having stated this background, the purpose of the present study is to directly determine the effects of acute stress on auditory sensitivity in a sample with different levels of EE. To achieve this goal, a combination of direct (uncomfortable loudness levels, ULL) and indirect (questionnaire) measures of hyperacusis was studied before and after an acute stress exposure. A common audiology test to assess hyperacusis is to determine uncomfortable loudness levels (ULL). Here we show for the first time a direct maladaptive reaction of acute stress on hearing problems. The consequence of this new finding highlights the importance of evaluating symptoms of long-term stress exposure in patients seeking help for hearing problems and vice versa.

## Methods

### Population and Design

**Figure 1:**
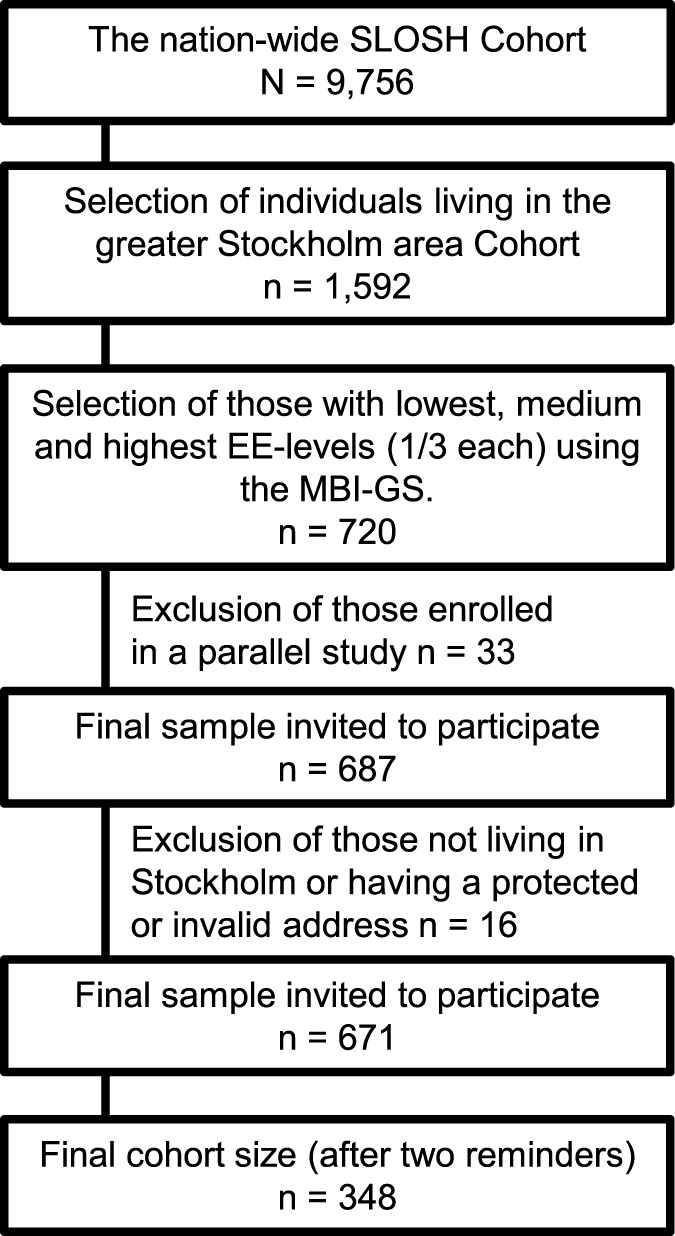
The flow of participants from the SLOSH cohort to the Stress and hearing study.

The present study is cross-sectional and includes subjective and objective measures of hearing, as well as subjective ratings of emotional exhaustion. The sample was drawn from the Swedish Longitudinal Occupational Survey of Health (SLOSH) [22], which was initiated by the Stress Research Institute at Stockholm University in 2006. The present cohort was established through the second data collection, which was conducted in April 2008 by Statistics Sweden. The sample of the present study was based on two types of inclusion criteria; 1) degree of EE and 2) living in the greater Stockholm area. The rationale for using the EE scores was previous findings indicating higher prevalence of hearing problems among individuals who are exposed to long-term stress [23], [24], [25]. For convenience and feasibility reasons, the participants had to be living in Stockholm County. The selection of participants was then based on scorings on the exhaustion dimension of the Maslach Burnout Inventory – General survey (MBI-GS). The reason for this was that it should be possible to study possible differences in hearing outcomes in relation to degrees of stress-related pathology. Consequently, the strategy was to select contrasting groups including the 200 women and men (100 each) with the highest EE scores, 200 with medium EE and 200 with the lowest EE scores. After plotting the EE scores for women and men separately, the cutoff was set around the highest quartile, the median and around the lowest quartile. The selection procedures yielded a sample of 720 individuals. Some of them enrolled in a parallel study were excluded. As a result, the final sample included 687 individuals consisting of 143 women and 127 men with low EE scores, 118 women and 110 men with medium EE scores and 119 women and 103 men with high EE scores. With the help of Statistics Sweden, these individuals were invited to participate in the study. After sending out the invitations, 16 participants were removed due to either not fulfilling the inclusion criterion of living in Stockholm or having a protected or invalid address. The final cohort size was thus 671. After two reminders, 348 (52%) individuals enrolled in the study. Figure 1 illustrates the flow of the participants.

The procedure of the study was described in an invitational letter, and was orally explained to each participant upon arrival. The study was approved by the Central Ethical Review Board in Stockholm (protocol no 2009/493-31/3). All gave their written consent to participate.

### Data Collection

Extensive questionnaires were used to assess the participants’ demographics and various dimensions of mental and physical health (depressive symptoms, sleep and recovery status and emotional exhaustion) and wellbeing (e.g. life satisfaction) as well as hearing status (e.g., tinnitus, hearing loss and hyperacusis) health behaviors (e.g. physical activity and smoking) and psychosocial work environment.

#### Hearing assessment

Hearing status was assessed in the laboratory using a pure tone audiogram (PTA, 500–8,000 Hz by octave steps) following the standard Hughson-Westlake procedure in both ears using circumaural headphones (TDH39). After that, a hearing-in-noise-test (HINT) [26], [27] was conducted in the left and right ears separately. Uncomfortable loudness levels (ULLs) were determined according to the SAME-method (see below for a further description). Each participant was instructed to let the examiner know when the pure tone became uncomfortably loud, by speaking into a microphone. The testing started at 1 kHz with a signal intensity of 70 dB. If this level was perceived as uncomfortably loud, the intensity was decreased by 10 dB and the starting level on the following frequencies was decreased to 60 dB. The sound intensity was increased in 5 dB steps until the participant gave a response that the sound was uncomfortably loud. The tested frequencies were 0.5, 1, 2 and 4 kHz. The left and right ears were tested separately. In three cases ULLs were erroneously measured only to 100 dB and in three cases only to 105 dB. Hyperacusis was also assessed using the Hyperacusis Questionnaire (HQ) [21].

Good hearing status was defined as having no hearing loss in any PTA frequency (i.e. ≤20 dB HL PTA). Exposure to noise at work was assessed by asking for the proportion of time study participants were exposed to weary noise (from 0 =  never to 6 =  all or almost all the time).

### EE Assessment

*Emotional exhaustion* was assessed with the Maslach Burnout Inventory general survey (MBI-GS) using the emotional exhaustion subscale [28]. The scale consists of five items, derived from the Maslach Burnout Inventory human services survey (MBI-HSS) in unmodified form. Scorings reach from 1 (every day) to 6 (a few times a year or less/never). The items included to assess the construct are: *“I feel emotionally drained by my work”, I feel completely exhausted when the working day is over”, “I feel tired when I get up in the morning to face a new working day”, “To work during a whole day is really stressful for me”, “I feel burned out of my work”.* Cronbach’s alpha and stability for the subscale have been reported to be satisfactory. Strong support for the construct validity of the Swedish translation of the MBI-HSS has been found [29]. The index was calculated on the basis that 4 out of 5 items had to be answered in order be included in the index.

#### Acute stress task

In order to maximize the likelihood of eliciting stress, the study participants were exposed to three stress-inducing tasks simultaneously. The first task consisted of an emotional Stroop-test [30], [31], [32], where participants were asked to identify the colors of rapidly alternating words on a computer screen. At the bottom of the screen were boxes with the words: blue, brown, grey, green, yellow, pink, red, black, and white, and the task was to click on the box corresponding to the color of the letters of the word currently displayed. In contrast to the traditional Stroop-test, emotionally charged and neutral words were used to elicit a greater stress response. The charged and neutral words were distributed equally and presented in random order. Examples of charged words were: death, hate, and enemy. Examples of non-charged words were rose, senior, and bread. Different interfering colors were blinking on the background of the screen, and random colors were concurrently presented by a speaker voice via headphones. The pace of the visual and auditory presentation was 30 words per minute, while the background color shifted 80 times per minute.

The second task, performed simultaneously, was a cold pressor exposure, i.e. hand in ice water (about 4°C), which may be more or less painful for different individuals. The non-dominant hand was inserted wrist-deep into a bowl of water and ice for the entire the Stroop-test, i.e. four minutes. The cold-pressor test has been extensively used in laboratory settings to elicit a stress response [33], [34], [35], [36]. It has been demonstrated that adding a social evaluative task further enhances the stress response [37]. Therefore, a social evaluation element was also included, i.e. video recording of the respondent and being observed by the researcher holding the camera. The video camera was stationed approximately 40 cm behind the computer screen, and participants were told that their facial expressions would be recorded for evaluation by a professional. The participants were instructed that they were allowed to remove their hand from the ice-water if the pain was unbearable. The recording started as soon as the headphones were properly equipped and one hand had been fully inserted the bowl of water.

#### Plasma samples for steroid analysis

Venous plasma samples were collected before and 20–30 minutes after the stress test. The samples were centrifuged and stored in a deep freezer in immediate conjunction with blood sampling at temperature −80 centigrades. They were analyzed with regard to steroid content by means of liquid chromatography tandem mass spectrometry [38]. The concentration of cortisol and estradiol was recorded for the purposes of the present study. Plasma cortisol concentration is an established stress (energy mobilization) indicator that is known to react within minutes after the onset of the stress exposure [39]. It is also known that the ability to respond with cortisol elevation may be hampered by conditions related to long lasting stress exposure i e stress lasting for weeks and months such as chronic fatigue [40]. It is also known that strong subjective reactions to a standardized stressor may be reduced by a pronounced cortisol reaction [41]. Estradiol is an anabolic hormone which protects against adverse effects of stress [42] . All blood samples were collected between 7.30 am and 11.30 am, between October and December 2009.

#### Statistical analyses

A one-way ANOVA with Bonferroni post-hoc tests was conducted in order to assess possible baseline differences in mean values of ULL. A 2-way ANCOVA was used to assess possible differences in mean ULLs over time (pre to post the acute stress task) between the EE-groups. Since there was a ceiling effect in the ULL assessment a new variable was created. If a person had a “>” symbol next to a 110 dB score, the value was set to 115 dB. This is a cautious approach to create at least a minor reduction in the ceiling effect. All the parametric analyses were conducted with this variable.

Multivariate analyses, proportional odds model (also called ordered logistic regression), were used to calculate possible odds ratios, including interacting or confounding effects of age, gender, ear wax and hearing loss (only PTA). All logistic regression analyses were adjusted for hearing loss and ear wax. For the logistic regression, ULLs were trichotomized. ULL values ≤85 dB HL were considered to be a sign of severe hyperacusis; 86–95 dB moderate hyperacusis and ≥96 normal values. EE was also divided by tertile split so that values between <1.2 was considered to be low levels of EE; 1.21 to 2.99 medium EE and ≥3 high EE. All p-values were 2-sided and significance was set at 0.05. Data analysis was performed using SPSS Statistics version 20 and SAS.

## Results

### Baseline and Post-stress Differences in ULLs between EE Groups

**Figure 2:**
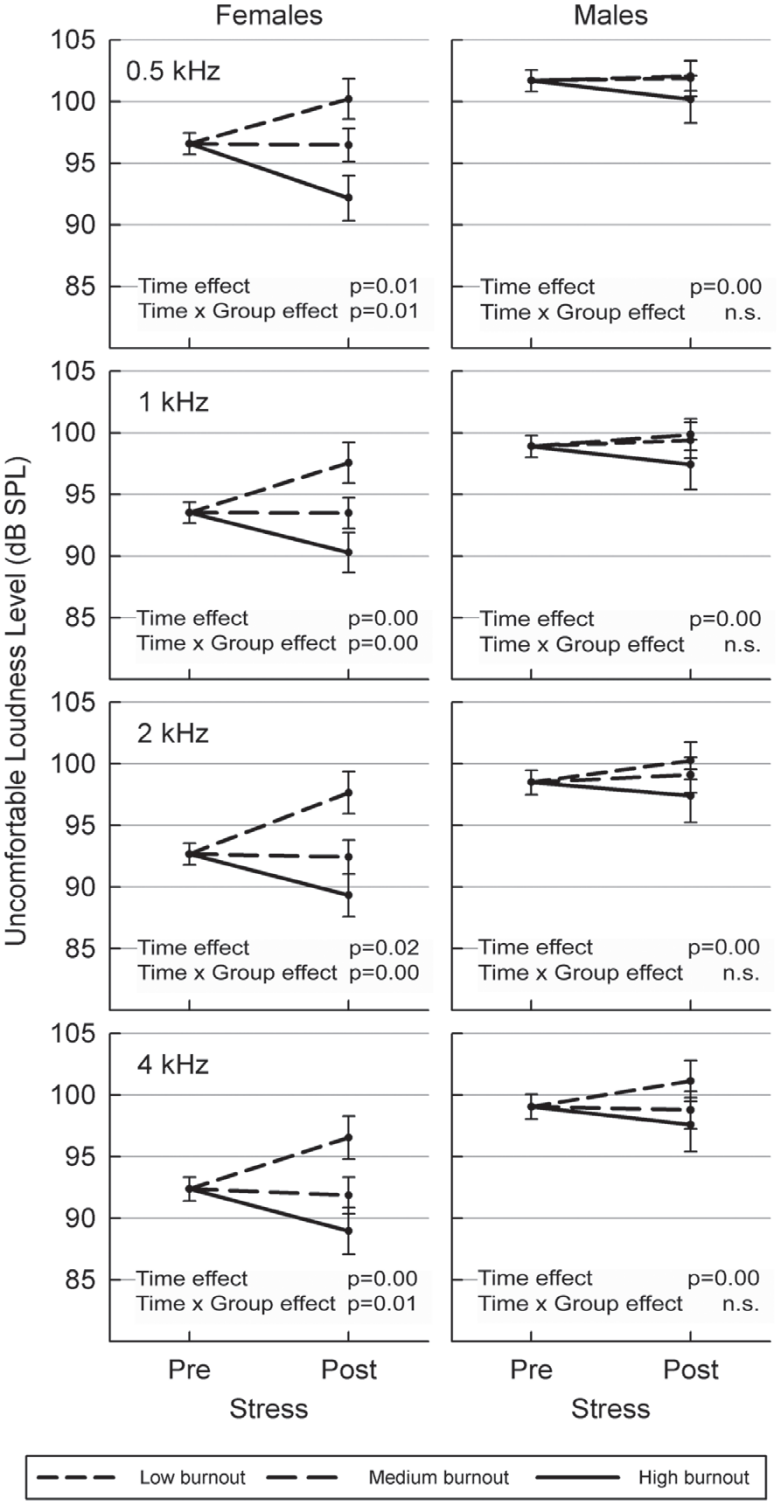
Mean (± SEM) uncomfortable loudness level thresholds before and after an acute stress task for females and males. Three groups differing in their emotional exhaustion levels (low, medium, high) are illustrated. The results from a two-way ANCOVA analyzing the right ear are shown.

There were no baseline differences in mean ULL levels between the three EE groups, except for the left ear 4 kHz where there was a mean difference of 6 dB between the low and high EE-groups (p = 0.035, one-way ANOVA with Bonferroni post-hoc test). However, after the acute stress exposure there were significant differences in mean levels of ULL (2 kHz right F = 5.78_df = 2 _p = 0.003; left F = 4.54_df = 2_ p = 0.011; 4 kHz right F = 5.25_df = 2_ p = 0.006, left F = 5.39_df = 2_ p = 0.005) between the EE-groups. Bonferroni post-hoc analyses showed that the differences in mean ULLs were between those with high vs. low EE. Mean differences between the lowest and highest EE quartile were on average 6.0 dB. Similar results were found for frequencies 0.5 and 1 kHz. In order to illustrate these findings a two-way ANCOVA was performed yielding significant differences between groups over time (Figure 2). The figure illustrates how women with high EE become more sensitive to pure tones at all frequencies tested after acute stress. In contrast, those with low levels of EE became more tolerant to higher levels of stimulation after acute stress. Due to ceiling effects (maximum sound level was 115 dB SPL) mean values do not give a perfectly correct picture of true dB differences. Still, differences between the EE groups post-stress are apparent. These differences would probably be even larger without the ceiling effect.

### Sex Differences

When the analyses were stratified for sex there were no differences in mean ULLs for men in different EE-groups, neither pre- nor post-stress. Women, however, only displayed a post-stress difference in mean ULLs and not a pre-stress difference, i.e. the exact same pattern as when analyzing the whole group consisting of both women and men. Thus, the differences that were found in the whole group are mostly influenced by the responses of the women. An independent samples t-test showed that there was a statistically significant sex-related difference in baseline ULLs for all frequencies (p<0.0001) where men had consistently higher thresholds than women. When analyses were stratified for EE, the sex-related difference remained for those with medium and high EE levels. For those with low EE, the differences were found on 1, 2 and 4 kHz for the right ear and 4 kHz for the left ear (p<0.05). Thus, it is clear that there are sex-related differences in ULLs, independent of EE status.

### Ceiling Effects

Due to safety precautions the maximum sound level used to elicit the ULL was set at 110 dB SPL (erroneously 105 dB for 3 individuals and 100 dB for 3 individuals; they were still included in the analyses). There were apparent ceiling effects among men, but the ceiling effect was less pronounced in women. Ceiling effects, i.e. 115 dB, were more common in men (19–28%) compared to women (10–16%) for the different frequencies. In order to determine if this was due to men having greater hearing loss and thus requiring higher sound intensities to elicit uncomfortable levels, a sex-stratified binary logistic regression was conducted on each ear separately. The dependent variable, ceiling effect, was coded as 0 =  No (i.e., <115 dB) and 1 =  Yes. The independent variable, hearing loss, was dichotomized on the basis that there was a hearing threshold of ≥20 dB on any frequency (each ear analyzed separately). With exception of the 2 kHz frequency, hearing loss did not increase the odds of having a ceiling effect in ULLs. For 2 kHz, men but not women, had higher odds of exhibiting a ceiling effect on the right (OR = 3.4, CI 1.1–10.4, p<0.05) and left ear (OR = 2.4, CI 1.0–5.6, p = 0.05). Thus, in general and with one exception (2 kHz) among men, worse hearing did not influence the ceiling effects in neither women nor men.

### Effects of Acute Stress

**Figure 3:**
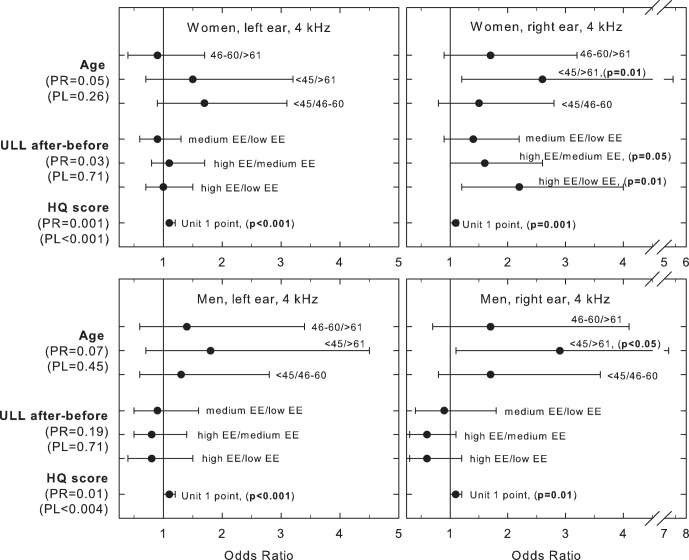
Ordered logistic regression assessing predictors of post-stress hyperacusis (ULL 4 kHz), including interacting or confounding effects of age, gender, ear wax and hearing loss (only PTA). All logistic regression analyses were adjusted for hearing loss and ear wax. PL (left) and PR (right) are overall tests of variables included in the estimated model.

**Figure 4:**
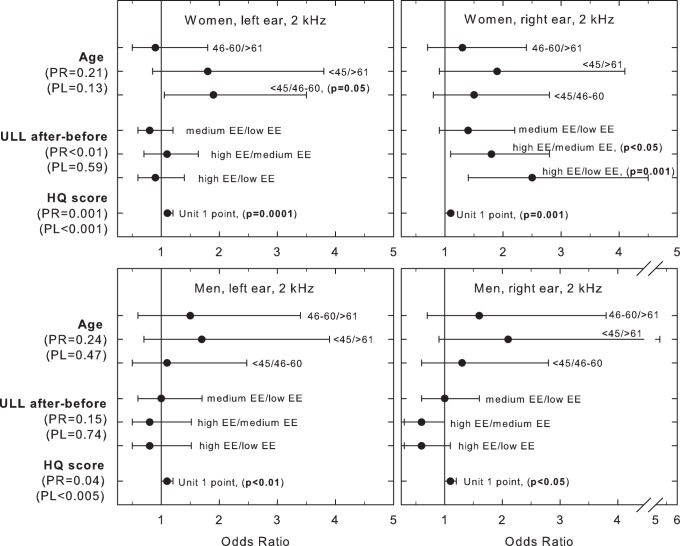
Ordered logistic regression assessing predictors of post-stress hyperacusis (ULL 2 kHz), including interacting or confounding effects of age, gender, ear wax and hearing loss (only PTA). All logistic regression analyses were adjusted for hearing loss and ear wax. PL (left) and PR (right) are overall tests of variables included in the estimated model.

The results demonstrate that women, but not men, who show symptoms of long-term stress display hyperacusis after the acute stress task. The odds of having hyperacusis were 2.5 (2 kHz, right ear; left ns) and 2.2 (4 kHz, right ear; left ns) times higher among those with high EE compared to those with low levels (Figures 3 and 4). When comparing those having high EE-levels with those displaying intermediate levels, the odds of having hyperacusis decreased to 1.8 (2 kHz, right ear; left ns) and 1.6 (4 kHz, right ear; left ns) times respectively. For both women and men, higher HQ-scores increased the odds of having hyperacusis in both ears (2 and 4 kHz; OR = 1.1, p<0.05). Among women (left ear 2 kHz), age (<45 years) significantly increased the odds of having hyperacusis (OR = 1.9, p<0.05). Men exhibited a similar pattern for the right ear 4 kHz (OR = 2.9, p<0.05) (Figures 3 and 4). All these results are adjusted for age, hearing loss and ear wax.

There is a systematic time*group effect for the different EE-levels on the right, but not left, ear for women (2 kHz χ^2^ = 10.07_df = 2_, p<0.01 and 4 kHz χ^2^ = 7.00_df = 2_, p<0.05). This demonstrates that the different EE groups develop differently over time with regard to ULL. There were no significant differences for the men at any frequency.

### Hormonal Changes after Acute Stress

The stress hormone cortisol (logarithmized) was assessed before and after the acute stress test. There was a significant time effect (two-way ANCOVA: All F = 28.093_df = 1_, p<0.0001; Men F = 22.040_df = 1_, p<0.0001; Women F = 10.586_df = 1_, p = 0.001), i.e. change over time in cortisol levels, for both women and men when adjusting for baseline cortisol levels, age and time of blood sampling. There was no significant time × group effect for the EE-groups when taking blood sampling time into account. This demonstrates that the different EE-groups did not change statistically differently over time. The cortisol levels decreased for all women. Additional statistical analyses were undertaken to understand the decrease in cortisol levels found for women. The findings showed that a variety of factors (age, estradiol levels, phase of the menstrual cycle, birth control pills and estrogen intake) confounds the cortisol response to stress in women. In response to acute stress, the cortisol changes in men increased. However, there were tendencies, albeit not statistically significant, showing that those with low and medium EE levels increased in cortisol concentration while those with high EE decreased (Table 1).

**Table 1:** The table shows the mean cortisol levels ± SD for men and women with low, medium and high EE, pre- and post the acute stress task.

EE level	Pre-stress cortisolMean (± SD) nmol/L	Post-stress cortisolMean (± SD) nmol/L	n
***Men***			***127***
Low	253 (±92)	271 (±107)	39
Medium	238 (±83)	253 (±95)	59
High	220 (±65)	217 (±53)	29
***Women***			***181***
Low	236 (±86)	207 (±86)	48
Medium	250 (±98)	236 (±97)	84
High	245 (±92)	229 (±89)	49

## Discussion

The salient finding of the present study was that women with high levels of emotional exhaustion had reduced thresholds to loudness (i.e. more sensitive) after the acute stress task. It is important to note that basal levels of ULLs did not differ between the EE groups and differences were only detected *after* the acute stress challenge. This means that the ULL test, when given to individuals with high levels of EE who are acutely stressed, will result in lower thresholds compared to individuals who are not emotionally exhausted. This also means that the ULL test will not detect signs of hyperacusis in women with high levels of EE if they are not acutely stressed during the testing. It cannot be expected that an acute stress task is performed in the clinics before and after ULL assessments and therefore other means of detecting hyperacusis in women with EE is needed. One way could be to question the patient about the circumstances that induce their hyperacusis. This novel finding highlights the importance of including EE in the diagnosis and treatment of hyperacusis, e.g. via questionnaires and interviews, since it is not considered standard procedure today.

The results also indicate a possible need to include stress management in the standard treatment procedures for hyperacusis. Clinical studies will be needed to confirm this suggestion, but the results from the present study indicate that this should be a high-priority treatment. In addition, findings from this and other studies confirm the importance of the association between symptoms of long-term stress and hearing problems [10], [23], [24], [43], [44], [45]. For example, EE was shown to be the factor with the strongest association with tinnitus [10].

### Sex Differences

Sex-related differences were apparent in baseline assessment of ULLs, where men had higher thresholds than women. It is well-known that, with increasing age, men have poorer hearing with PTAs compared to women until post-menopausal age [46] and this could be an underlying factor for the baseline differences in ULL. In our sample there was a statistically significant sex-related difference in PTA at 4 kHz, where men had on average a 6 dB higher threshold (p<0.01). We have previously found that women and men with different levels of EE exhibit diverse patterns of hearing problems [10], [24]. It has been shown that men and women systematically display higher prevalence of hearing problems with increasing EE levels [24]. However, in women with high EE, the prevalence increase is more pronounced than in men. This may indicate that sex-related biological differences exist and may constitute an underlying cause of hearing problems.

Post-menopausal women show an accelerated hearing loss, suggesting that sex hormones may play a role [46]. In fact, women showing an increase in estradiol after acute stress have 2.35 times higher odds of hyperacusis compared to those who showed a decrease (right ear; 4 kHz). An increase in relation to unchanged levels yielded 1.99 higher odds of hyperacusis. However, a decrease in relation to unchanged levels did not yield any significant change in odds of having hyperacusis. These data are not presented in the results section due to the fact the effects were only significant for the right ear in women and only for 4 kHz. The most likely reasons for this limited statistical significance of estradiol may be the large variation of hormone values in the sample or a lack of power. Mean (± SD) estradiol levels for women in the different EE-groups were as follows: Low EE 142.2±264.1 pmol/L; Medium EE 156.3±265.2 pmol/L; High EE 197.5±324.9 pmol/L.

### Limitations

One limitation is that only 52% of the invited participants enrolled. This possible selection bias can make the results less generalizable to the general population. However, the participants were selected from a cohort that has been specially compiled to be representative of the general Swedish population. Therefore, the risk for selection bias is somewhat reduced. A strength is that the selection of participants was based on different levels of EE. This affords the opportunity to contrast these groups, which makes it possible to directly test our hypotheses on a lower number of participants. Another limitation is that classification cut-offs for the logistic regression analyses may influence the results. Considering the ceiling effect and variations in normal distribution of data it is not possible to only rely on parametric statistical methods. Therefore, both parametric and non-parametric analytic strategies were adopted and yielded similar results. This indicates that the classifications of variables were reasonable.

### Hormonal Response to Stress Task

There are often uncertainties when analyzing hormones due to behaviors of the study participants that may confound the results. These confounders could include not fasting before blood sampling (despite being requested to fast), smoking, recent stressful life events, medications and environmental factors in the clinic. The confounding effects of seasonal variations in hormone levels were counteracted by limiting the time of the study from October to December. Furthermore, the diurnal variations were limited as much as possible by only collecting blood samples between 7 and 11.30 am. Cortisol is known to have large variations particularly in the morning hours and this was also noted among the participants in this study.

The analyses of plasma cortisol concentration before and after stress exposure were performed for confirmation that biological stress had been elicited. This was only partly confirmed in the sense that men with low and medium EE-scores showed tendencies of increased cortisol levels, while those with high EE-scores showed a blunted cortisol response. This is consistent with the idea that subjects with fatigue have a reduced ability to respond with cortisol excretion in an acute stress situation [40] and also consistent with the finding that after the stress exposure men with high EE-scores are more disturbed [41] by the situation, which is also mirrored in stronger reactions to noise. In the present study, there were no statistically significant changes in auditory sensitivity (ULL) for men with different EE-levels. This may be due to a relatively low number of men, yielding insufficient statistical power. However, as illustrated in Figure 2, the trend was similar among men and women and a larger sample may have yielded a statistically significant result. Another possible explanation is that men may be less sensitive to pain compared to women [47], [48]. If so, the cold pressor test may have elicited less pain and thereby less stress among the men, yielding less pronounced results with regards to the ULLs. For men, there was a tendency (not statistically significant) for cortisol to increase after stress among those with low and medium EE-levels and decrease for those with high levels. The cortisol levels however decreased for all groups of women in the study. This may be due to more confounding factors in the female group, such as intake of estrogen, menstrual cycle phase, contraceptive pills, etc. However it should be pointed out that [49] the Stroop test – even in less severe forms than the one used in the present study – is a strong stimulus for increased plasma concentration of catecholamines. In this particular study we also added the cold pressor test. These two tests together should be regarded as a very strong laboratory stressor with pronounced ability to raise sympathoadrenomedullary activity. Plasma cortisol has a pronounced circadian variation with sharp decreases during the morning hours. The apparent (cortisol) decrease observed particularly in women may be confounded by the strong circadian variation and other factors mentioned above, which we have not been able to fully adjust for. In summary, the stressors used in this study resulted in men showing the expected rise in cortisol after acute stress whereas the response of women may have been confounded by factors not directly related to the experiment.

### Conclusion

For the first time it is shown that EE is a significant factor to consider for a complete diagnosis and effective treatment of hyperacusis, particularly in women. Patients seeking help for hyperacusis, but exhibit normal ULLs, should also be assessed for emotional exhaustion for a correct diagnosis. Hyperacusis is a multi-dimensional phenomenon and co-morbidities need to be taken into account. The underlying reasons for the sex-related differences are not understood and additional studies are needed to identify explanatory factors.
