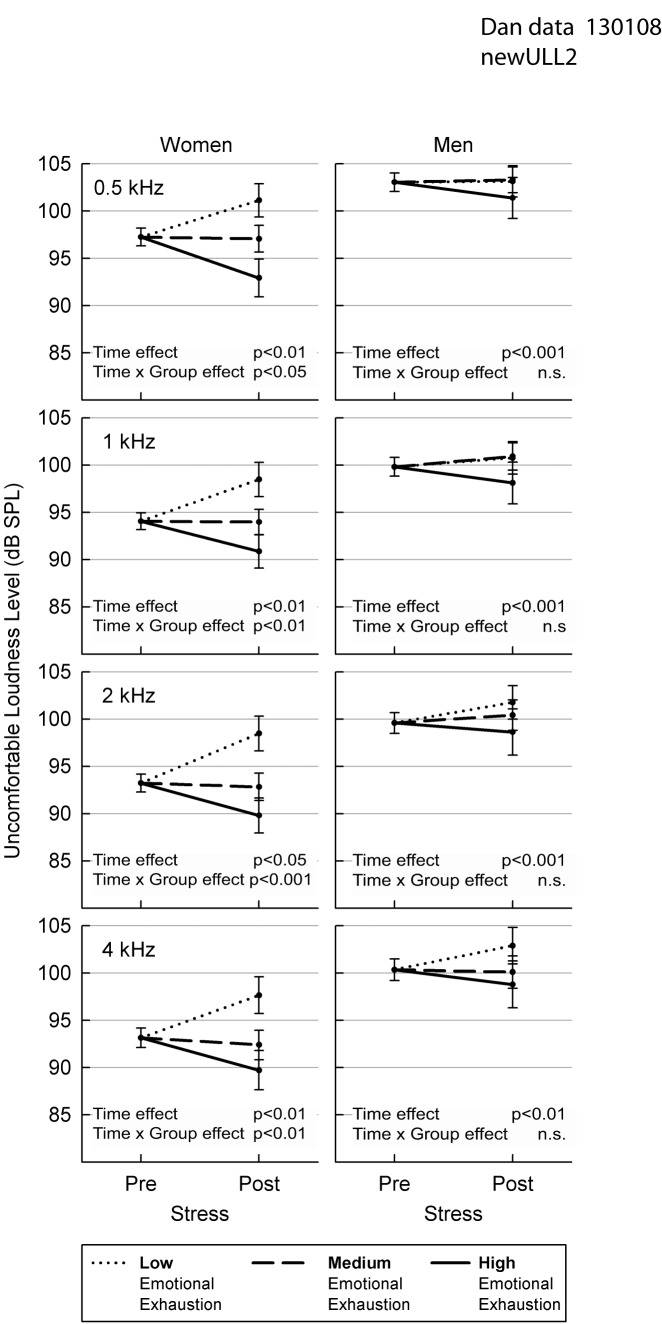# Correction: Acute Stress Induces Hyperacusis in Women with High Levels of Emotional Exhaustion

**DOI:** 10.1371/annotation/6a09a2e1-5c83-4ae7-859b-454de3e21814

**Published:** 2013-04-16

**Authors:** Dan Hasson, Töres Theorell, Jonas Bergquist, Barbara Canlon

There was an error in Figure 2. Please see the correct Figure 2 here: 

**Figure:**